# Characterization of the HollandPTC proton therapy beamline dedicated to uveal melanoma treatment and an interinstitutional comparison

**DOI:** 10.1002/mp.15024

**Published:** 2021-07-11

**Authors:** Emmanuelle Fleury, Petra Trnková, Kees Spruijt, Joël Herault, Franciska Lebbink, Jens Heufelder, Jan Hrbacek, Tomasz Horwacik, Tomasz Kajdrowicz, Andrea Denker, Anaïs Gerard, Petter Hofverberg, Maria Mamalui, Roelf Slopsema, Jean‐Philippe Pignol, Mischa Hoogeman

**Affiliations:** ^1^ Department of Radiotherapy Erasmus MC Cancer Institute, University Medical Center Rotterdam The Netherlands; ^2^ Holland Proton Therapy Center Delft The Netherlands; ^3^ Departement of Radiation Oncology Medical University of Vienna Vienna Austria; ^4^ Departement of Radiation Oncology Centre Antoine Lacassagne Nice France; ^5^ Helmholtz‐Zentrum Berlin für Materialien und Energie Berlin Germany; ^6^ Department of Ophthalmology Charité ‐ Universitätsmedizin Berlin Berlin Germany; ^7^ Paul Scherrer Institute Center for Proton Therapy Villigen Switzerland; ^8^ Institute of Nuclear Physics Polish Academy of Sciences Kraków Poland; ^9^ Department of Radiation Oncology University of Florida Gainesville Florida USA; ^10^ Department of Radiation Oncology Emory Proton Therapy Center Atlanta Georgia USA; ^11^ Department of Radiation Oncology Dalhousie University Halifax Canada

**Keywords:** eyeline, proton therapy, uveal melanoma

## Abstract

**Purpose:**

Eye‐dedicated proton therapy (PT) facilities are used to treat malignant intraocular lesions, especially uveal melanoma (UM). The first commercial ocular PT beamline from Varian was installed in the Netherlands. In this work, the conceptual design of the new eyeline is presented. In addition, a comprehensive comparison against five PT centers with dedicated ocular beamlines is performed, and the clinical impact of the identified differences is analyzed.

**Material/Methods:**

The HollandPTC eyeline was characterized. Four centers in Europe and one in the United States joined the study. All centers use a cyclotron for proton beam generation and an eye‐dedicated nozzle. Differences among the chosen ocular beamlines were in the design of the nozzle, nominal energy, and energy spectrum. The following parameters were collected for all centers: technical characteristics and a set of distal, proximal, and lateral region measurements. The measurements were performed with detectors available in‐house at each institution. The institutions followed the International Atomic Energy Agency (IAEA) Technical Report Series (TRS)‐398 Code of Practice for absolute dose measurement, and the IAEA TRS‐398 Code of Practice, its modified version or International Commission on Radiation Units and Measurements Report No. 78 for spread‐out Bragg peak normalization. Energy spreads of the pristine Bragg peaks were obtained with Monte Carlo simulations using Geant4. Seven tumor‐specific case scenarios were simulated to evaluate the clinical impact among centers: small, medium, and large UM, located either anteriorly, at the equator, or posteriorly within the eye. Differences in the depth dose distributions were calculated.

**Results:**

A pristine Bragg peak of HollandPTC eyeline corresponded to the constant energy of 75 MeV (maximal range 3.97 g/cm^2^ in water) with an energy spread of 1.10 MeV. The pristine Bragg peaks for the five participating centers varied from 62.50 to 104.50 MeV with an energy spread variation between 0.10 and 0.70 MeV. Differences in the average distal fall‐offs and lateral penumbrae (LPs) (over the complete set of clinically available beam modulations) among all centers were up to 0.25 g/cm^2^, and 0.80 mm, respectively. Average distal fall‐offs of the HollandPTC eyeline were 0.20 g/cm^2^, and LPs were between 1.50 and 2.15 mm from proximal to distal regions, respectively. Treatment time, around 60 s, was comparable among all centers. The virtual source‐to‐axis distance of 120 cm at HollandPTC was shorter than for the five participating centers (range: 165–350 cm). Simulated depth dose distributions demonstrated the impact of the different beamline characteristics among institutions. The largest difference was observed for a small UM located at the posterior pole, where a proximal dose between two extreme centers was up to 20%.

**Conclusions:**

HollandPTC eyeline specifications are in accordance with five other ocular PT beamlines. Similar clinical concepts can be applied to expect the same high local tumor control. Dosimetrical properties among the six institutions induce most likely differences in ocular radiation‐related toxicities. This interinstitutional comparison could support further research on ocular post‐PT complications. Finally, the findings reported in this study could be used to define dosimetrical guidelines for ocular PT to unify the concepts among institutions.

## 1 INTRODUCTION

Proton therapy (PT) is a well‐established treatment modality for patients with uveal melanoma^1^ (UM), including choroidal, iris, or ciliary body tumors. Reported clinical outcomes show a 5‐yr local tumor control rate over 95% with an eye retention rate of 90%.^2^, ^3^, ^4^, ^5^, ^6^, ^7^, ^8^, ^9^, ^10^ A dedicated ocular nozzle mounted on a horizontal passive scattering beamline is used for the treatment in most facilities. Its properties are adapted to the needs of small superficial high‐dose delivery in the shortest time possible. Small‐field irradiation, sharp penumbrae and high‐dose rate to limit patient treatment time are thus the physical and dosimetrical pivotal requirements for a proton eyeline. Only a few centers were designed with a low‐energy accelerator with initial extraction energy up to 70 MeV resulting in optimal beam characteristics for the treatments of small UM located below 4.00 g/cm^2^ in depth.

As the number of proton facilities steadily increases worldwide, so does the number of dedicated proton eye treatment rooms, which has recently reached 20.^11^, ^12^ Most of them are integrated within a multiroom PT center, connected to a high‐energy accelerator generating beams with initial extraction energy up to 250 MeV corresponding to a proton range of more than 30.00 g/cm^2^ in depth. Hospital‐based cyclotrons remain the most widely used accelerators in ocular beamlines. They produce high‐dose rates while keeping a short treatment time close to 1 min. In recent years, PT has been drastically moving from passive scattering towards active beam scanning. Collimated scanning proton beam modalities connected to a cyclotron and synchrotron‐based facilities have been reported in research^13^, ^14^ and in clinical setting.^15^, ^16^

The first Varian (Varian Medical Systems Particle Therapy GmbH & Co. KG, Troisdorf, Germany) cyclotron‐based proton eyeline was installed at HollandPTC (Delft, The Netherlands) in 2018, and ocular treatments started early 2020. Patients with a medium to a large tumor (Tumor Node Metastasis staging form, American Joint Committee on Cancer [AJCC], updated Eighth edition^17^) or with a critical posterior tumor abutting the optic disc within less than 2 mm from the tumor edge are eligible for the therapy. It has been estimated that 50 patients a year will be treated with the HollandPTC ocular beamline.^18^ The clinical workflow^19^, ^20^, ^21^, ^22^, ^23^, ^24^ is similar to other existing institutions. Three to five tantalum clips surrounding the transilluminated tumor base are sewn onto the sclera during surgery 2 weeks before the PT treatment. The clips are needed for tumor location, treatment planning, setup, and treatment position verification. For four decades, the proton dose planning has been performed using a generic geometrical tumor and organs‐at‐risk models^25^ created based on surgical caliper measurements, ultrasound measurements, eye fundus photography, and any other 2D ophthalmologic information.^26^, ^27^, ^28^ A set of orthogonal X‐ray images showing the clip positions is used to select the most suitable gazing angle for the therapy plan. The patient physical ability is also considered. Additionally, at HollandPTC, dedicated ocular MRI protocols^29^, ^30^, ^31^, ^32^ to assess additional volumetric anatomy^33^, ^34^ and functional^35^ information of the tumor have been introduced as part of the clinical workflow.^29^, ^30^, ^31^, ^32^ A total prescribed relative biological effectiveness (RBE) equivalent dose of 60 Gy is delivered in four consecutive days. A constant RBE value of 1.1 is used. Patient eye positioning is verified by matching the clips with the 2D X‐ray images. A point‐like light source indicates a gazing direction to a patient. A real‐time camera monitors the stability of the gazing angle.

Currently, there are no international guidelines on the dosimetrical performance of dedicated ocular beamlines. Thefore, existing centers usually follow an internally developed protocol that may differ from a protocol used at another institution. Moreover, new centers have to rely on close collaboration with existing institutions. This study aimed to report on the design, physical, and dosimetrical properties of the first ocular PT beamline developed by Varian and compare the results against five existing institutions treating UM for many years. The institutions were chosen to represent a wide selection of installation approaches. We focused only on dedicated eye nozzles with a passive scattering system. The six centers in this study represent different combinations of cyclotron designs, nozzle components, and manufacturers. The impact of the dosimetrical differences was evaluated by simulation of seven tumor‐specific case scenarios to investigate to what extent these differences affect in‐depth dose distributions, and therefore, radiation‐related toxicity outcomes.

## 2 MATERIALS AND METHODS

### 2.1 Description of the HollandPTC ocular beamline

#### 2.1.1 Beam delivery system

A horizontal fixed passive scattering beamline dedicated to the treatment of ocular lesions uses the compact Varian superconducting cyclotron. The initial energy of the near‐monoenergetic proton beam is 250 MeV as the same cyclotron delivers the beam to two pencil beam scanning gantries and one fixed beam research line. The proton beamlet first passes through an energy selection system comprising a degrader and an energy slit located straight downstream the cyclotron. The double‐wedge degrader made out of a low‐Z material (carbon) reduces the incident energy while limiting the scattering of secondary protons. It, however, introduces energy and angular spread, which are then transported throughout the optical magnetic beam pipe. The adjustable achromatic energy slits are placed downstream of the degrader group to improve beam properties. They can optically trim away the polluting energies by moving the copper blocks in and out of the optical beam pipe. The closing of the energy slits results in a decreased distal dose fall‐off (DDF). If the opening of the energy slits is larger, the transmission is increased and shorter treatment times are achievable. An optimal position of the slits was defined to achieve a trade‐off between treatment time and beam quality. This position is kept constant to maintain the same beam properties for all treatment indications. TDipoles and quadrupoles adjust the proton beam envelope in the beam transport system, where additional bending and focusing are applied. Finally, the beam strikes the entrance of the ocular room with a low fixed kinetic energy of 75 MeV, an energy spread of 1.10 MeV (simulated from the pristine Bragg peak), and a beam current of 4 nA. The minimum dose rate is 13.6 Gy/min at the isocenter.

#### 2.1.2 Varian eye nozzle components

**FIGURE 1:**
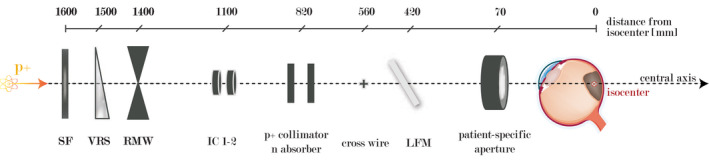
A schematic drawing of the HollandPTC eye nozzle, including its main components with respect to their position. SF, scattering foil; VRS, variable range shifter; RMW, range modulator wheel; IC, set of two ionization chambers; p+, proton; n, neutron; LFM, light field mirror. Not to scale. [Color figure can be viewed at wileyonlinelibrary.com]

The fixed horizontal eye nozzle at HollandPTC is a single scattering system. It has a length of 153 cm. The central axis of the beam is located 150 cm above the floor. The nozzle contains the following elements: a scattering foil, a variable range shifter, a selected range modulator wheel (RMW), a pair of ionization chambers (IC1‐2), a proton collimator, a neutron absorber, a cross wire, a light field mirror, and a snout for patient‐specific apertures (Figure 1). A scattering foil made of 250‐µm‐thick tantalum is the first integrated element located approximately 1600 mm away from the isocenter. It ensures a flat lateral dose distribution at the beam isocenter by spreading the field laterally to a therapeutic size. Currently, only one scattering foil is used clinically at HollandPTC. The scattering foil is considered as the virtual source. A motorized wedge‐shaped variable range shifter wheel with increasing thickness of Lexan™ Polycarbonate PC‐1000 (physical thickness from 0.20 to 3.75 cm) is used to pull back the range to a required penetration depth (residual range) based on the most distal edge of the treated target. Following Lexan™ RMW creates a spread‐out Bragg peak (SOBP) in the longitudinal direction corresponding to the largest basal diameter of the treated volume. During irradiation, the wheel spins with a frequency of 1200 rotations per minute, resulting in an oscillation of the beam range in tissue and thus delivers nearly simultaneous dose at several depths. A set of RMWs of different water equivalent depth (WED) modulation widths ranging from 0.60 g/cm^2^ (smallest SOBP) to a full modulation (3.97 g/cm^2^) is available. The given set generates any SOBP in discrete steps of 0.30 g/cm^2^ or less over the entire treatment range. An appropriate combination of a variable range shifter value with a specific RMW is required to generate a homogeneous SOBP at a certain depth.

The beam intensity is continuously controlled by an ionization chamber system located in the nozzle. It contains a set of two independent PTW air‐vented ionization chambers (PTW, Freiburg, Germany, IC1‐2). Each chamber has an area density of 19.4 mg/cm² and comprises three sections: two dose monitoring sections for detecting any deviation of absorbed dose or dose rate and one segmented section divided into four quadrants for measuring the symmetry of the transverse dose distribution. A proton collimator (brass, circular 28‐mm inner diameter) located between the beam modifying elements and the set of two ionization chambers absorbs the protons scattered off in the lateral direction. It is followed by a neutron absorber (10‐cm‐thick ultrahigh molecular weight polyethylene, circular 38‐mm inner diameter). A crosshair made of two orthogonal 0.30‐mm copper wires is aligned to the central axis of the beam, which is located downstream of the ionization system. The next element is a dimmable light field mirror system (gold‐coated Kapton) containing a LED to indicate a straight gazing angle being reflected onto the sclera.

At the end of the nozzle, a 25‐cm‐long snout consisting of a tube with a diameter of 40 mm is mounted. The maximum circular clinical field size (FS) at the beam isocenter is approximately 35 mm (FS‐35 mm). A brass aperture in a required shape designed with either a circular or tumor‐specific geometry can be mounted onto the snout to provide final lateral beam shaping. The thickness of the aperture is 10 mm to stop the protons of the highest energy available in the eye treatment room.^36^ Patient‐specific aperture is shaped by an in‐house milling machine (PAR SCIENTIFIC A/S, Odense S, Denmark) to the beam's eye view tumor projection. The patient‐specific aperture can be rotated (up to ±10°) on the day of the treatment depending on the torsion of the eye.

Outside of the nozzle, there are two different systems to manage gazing direction position and monitoring. The first one is a fixation LED light mounted onto a rotatable bar for guiding the patient to stare at the defined position. For small gazing angles, an add‐on LED is necessary. The second system is three CCD cameras that can be angulated differently. They monitor the fixation of the eye during the irradiation in real time.

#### 2.1.3 Varian eye treatment room

The patient is immobilized in a seated position with an individualized thermoplastic mask (Qfix, Aquaplast BoS RT1882, Avondale, PA, USA) with a bite block (Orbis A silicon putty) integrated with a head frame. The same type of head frame is also used for mounting quality assurance devices. The treatment chair is anchored to the floor on a rail system and aligned to the central beam axis. The nominal IEC testing limit of the chair is 135 kg. The chair can be moved along four linear (upwards/downwards, left/right, along the beam direction and adjustment in patient's height) and two rotational axes (tilting head backward/forward and rotating the chair left/right). The X‐ray imaging system with two amorphous silicon orthogonal flat imager panels (PaxScan 1313DX, Varian Medical Systems Particle Therapy GmbH & Co. KG, Troisdorf, Germany) acquires anterior and lateral radiographs. Following the IEC 61217 standard definition, the beam isocenter located along the central axis at 70‐mm downstream the outermost part of the final patient‐specific aperture coincides with the imaging one. The isocenter and the beam axis are visualized by a system of lasers. The distance between the chair and the nozzle can be adjusted to place the center‐of‐mass of the target at the isocenter.

### 2.2 Dosimetrical properties

#### 2.2.1 Dosimetry under non‐reference conditions

Depth dose distributions on the central axis and lateral dose distributions were measured using a mini MP3‐XS Perspex water tank (PTW, Freiburg, Germany). Its inner dimensions were 19.60 cm × 19.40 cm × 19.50 cm, and the water equivalent thickness of the entrance window was 0.28 g/cm^2^ (tolerance ±0.003 g/cm^2^). The inner surface of the front side of the water tank was aligned to the lasers, in both lateral and vertical directions.

Percentage depth dose distributions (PDDs) were measured with an Advanced Markus parallel‐plate chamber (PTW, TN34045) with a 0.106‐g/cm^2^ water equivalent cap thickness and a sensitive volume of 0.02 cm^3^. The chamber was mounted on the chamber holder (PTW, Trufix) and aligned with the central axis. The scanning started at the position where the chamber touches the inner surface of the entrance window of the water tank corresponding to a measurement depth of 0.47 g/cm^2^ (sum of the water equivalent thickness of the reference chamber, the entrance window of the water tank, and the cap of the Advanced Markus chamber). The scanning motion reproducibility was ±0.10 mm. A large‐sized plane parallel reference ionization chamber (PTW, TM7862) was positioned at the outer surface of the phantom entrance window. The PTW Tandem XDR dual‐channel electrometer was connected to the TBA control unit (PTW, T41013). The PTW MEPHYSTO mc^2^ software was used for reading out the measurements.

Lateral profiles of various circular FSs ranging from 10‐ to 35‐mm diameters were measured using a microdiamond single‐crystal diamond detector (PTW, T60019) with a sensitive volume of 0.004 mm^3^.

Both PDDs and lateral profiles were measured according to the International Commission on Radiation Units and Measurements (ICRU) Report No. 78.^37^ The SOBP widths were defined as the distance between 90% of proximal and distal doses (p90% and d90%, respectively). The curves were normalized at the average value within the treatment field, that is, the dose region that encompassed the SOBP width minus two DDFs from the distal 50% and one DDF from the proximal 90%. The DDF was defined as the distal 80%/20% dose gradient. The lateral profiles were normalized to the maximum dose. The LP for collimated broad divergent beams was defined within the 80%/20% width. Symmetry and flatness of the transverse profiles as a function of FS were analyzed at the middle of the fully modulated SOBP following the ICRU Report No. 78 recommendations. The flatness was established within the treatment field region, therefore avoiding the dose increase at the edges of the fields.

#### 2.2.2 Dosimetry under reference conditions

The absorbed dose determination in water under reference conditions (SOBP_REF_, FS_REF_, and z_REF_) followed the International Atomic Energy Agency (IAEA) Technical Report Series (TRS)‐398 Code of Practice for protons beams^38^ and the ICRU Report No. 78.^37^ The same Advanced Markus chamber (PTW, TN34045) and the setup as previously described were used. The chamber coupled to a PTW Unidos E T10010 electrometer was independently calibrated in a ^60^Co beam as reference beam quality Q_0_. The reference depth z_REF_ was defined at the middle of the full modulation width (SOBP_REF_ = 3.97 g/cm^2^). As the combined WED of the reference chamber, the water tank window, and the cap of the Advanced Markus chamber was 0.47 g/cm^2^, the z_REF_ in water was equal to 1.52 g/cm^2^. The air gap between the exit of the nozzle and the entrance window of the water tank was 70 mm. The variable range shifter was removed from the beam path to reach the maximal penetration. A FS‐35 mm was used to define the reference FS (FS_REF_). The readings were corrected according to the IAEA TRS‐398 protocol for actual temperature and pressure, voltage polarity, and ion recombination effects. Dose per monitor unit (Dose/MU) as a function of FS (FS_i_, SOBP_REF_, and z_REF_) and SOBP width (FS_REF_, SOBP_i_, and z_1/2_) was determined on the central axis of the proton beam. The results were normalized to the (Dose/MU)_REF_.^39^ The estimated standard uncertainty in mGy/MU determination with the Advanced Markus chamber is 2.30%.^15^, ^38^, ^40^

#### 2.2.3 SAD and effective source size

For the virtual surface‐to‐axis distance (SAD) determination, lateral profiles of three SOBP widths (0.60, 1.10, and 2.00 g/cm^2^) were measured in air at the isocenter and distances 100, 200, and 400 mm from isocenter with EBT3 Gafchromic films (Ashland ISP Advanced Materials, NJ, USA). A FS‐35 mm was used. For each profile, the full‐width half‐maximum (FWHM) was determined. The virtual SAD was obtained as an average of the three linear fits of the FWHMs as a function of the distance from the isocenter. The effective source size was calculated from the LP behavior (Figure 4D).^41^, ^42^ The effective source corresponds to the angular finite source size seen at the isocenter, which combined the effects of the incident beam size with the scattering system.

#### 2.2.4 Secondary neutrons

Equivalent neutron dose due to beam contamination was assessed with the FHT 762 Wendi‐II neutron dose rate detector connected to the digital Geiger counter FH 40 G survey meter (Thermo Fisher Scientific™, Inc.) for read‐out. Measurements were performed under reference conditions. The equivalent neutron dose was estimated at three body locations corresponding to the (i) contralateral half brain (10‐cm lateral shift from isocenter), (ii) chest region (35‐cm downward shift), and (iii) abdominal region (50‐cm downward shift). Overall estimated uncertainty in neutron dose is 20%.^43^

### 2.3 Comparison with other eyelines

#### 2.3.1 A Geant4‐based energy spread determination

Energy spread values for all the centers were assessed by Monte Carlo simulations using Geant4 code version 4.10.06 and the physics list source file QBBC (CERN, Geneva, Switzerland). A nondivergent proton beam was generated homogeneously over a 60‐mm‐diameter disc and propagated in water. The simulated depth dose distributions were sampled over a 10‐mm‐diameter surface at 0.02‐g/cm^2^ depth intervals. Pristine Bragg peaks were simulated for nominal beam energies of 62.5, 68, 70, 75, and 104.5 MeV corresponding to the energy at the ocular nozzle entrance at CAL, Nice; HZB‐Charite, Berlin; both IFJ PAN, Krakow and CPT PSI, Villigen; HollandPTC and UFHPTI, Florida, respectively. The simulated pristine Bragg peak for each center was individually matched to its corresponding measured one by fine‐tuning the energy spread value and a trial‐and‐error method. The resulting energy spread values took into account the energy straggling effect in the energy selection system.

#### 2.3.2 Eyeline properties and clinical indications

Five ocular proton facilities using different technologies and beam energies were included in this study. All institutions provided a set of distal, proximal, and lateral region measurements. The results were compared against the HollandPTC ocular beamline properties. The summary of the technical characteristics of the different installations is provided in Tables 1 and 2. An overview of all the measurement tools used at each institution is listed in Table 3. The last two columns of Table 2 present the limiting values of tumor height (TH) and largest tumor basal diameter (LBD) in each institution. The patients defined within the criteria will be indicated for PT treatment.

**TABLE 1:** Specifications of dedicated ocular facilities included in this study: technical design.

	Cyclotron		Eye nozzle					In‐room beam characteristics			
	Type	Extracted energy (MeV)	Type	Eyeline energy (MeV)	Scattering system	Scattering foil(s)	Range shifter	Energy spread at the nozzle entrance Geant4 simulations (MeV)	Dose rate (Gy/min) at isocenter		Treatment irradiation time (seconds/fraction for 15 Gy(RBE)^c^)
HZB‐Charite, Berlin	Cyclotron, Scanditronix with HZB design	72	In‐house HZB	68 (max. 72)	Single	Ta 50 µm	PMMA	68 ± 0.20	13 to 30	→	30 to >60
UFHPTI, Florida	Cyclotron, IBA	230	IBA	104.5	Single	None	PC	104.5 ± 0.70	Min. 20	→	<40
HollandPTC	Cyclotron, Varian	250	Varian	75	Single	Ta 250 µm	PC	75 ± 1.10	Min. 13.6	→	<60
IFJ PAN, Krakow	Cyclotron, IBA	235	In‐house	70	Single	Ta (25 + 25) µm	PMMA	70 ± 0.70	Min. 12	→	<70
CAL, Nice	Cyclotron, in‐house	65	In‐house	62.5	Single	Ta 50 µm	PMMA	62.5 ± 0.10	0.1 to 100	←	10
CPT PSI, Villigen	Cyclotron, Accel/Varian	230	In‐house, Accel/Varian	70	Multiring^a^	Ta + PC^b^	PC	70 ± 0.70	Min. 15	→	<60

Abbreviations: PC, polycarbonate; PMMA, polymethyl methacrylate; RBE, relative biological effectiveness; Ta, Tantalum.

^a^ Multiring double scattering system.

^b^ Mixture of Ta and PC for the set of nine clinically used scattering foils.

^c^ Biological dose.

**TABLE 2:** Specifications of dedicated ocular facilities included in this study: beam parameters and clinical ocular PT indications.

	Snout‐isocenter distance (mm)	Average virtual SAD (cm)	Effective *σ*_source size_ (mm)	Maximum commissioned range in water (g/cm^2^)	Range of modulation widths WED (min; ∆; max) (g/cm^2^)	Indications for ocular PT	
						Tumor height (mm)	Largest tumor basal diameter (mm)
HZB‐Charite, Berlin	70	350	~2.00	3.14	(0.60; >0.10; 3.20)	1.00 to 17.10	2.00 to 25.00
UFHPTI, Florida	70	169	9.20	3.40	(0.50; 0.07 to 0.30; 3.40)	1.00 to 13.00	5.00 to >20.00^a^
HollandPTC	70	120	4.40	3.97	(0.60; <0.30; 3.97)	>7.00^b^	>15.00^b^
IFJ PAN, Krakow	70	165	12.50 (at SAD)	3.15	(0.60; 0.20; 3.15)	1.40 to 14.50^c^	6.00 to 22.00^c^
CAL, Nice	70	300	~0.10	3.20	(0.20; 0.50; 3.20)	0.50 to 20.00	2.00 to 25.00
CPT PSI, Villigen	70	175	*x* = 8.00 *y* = 18.00	3.51	(0.77; 0.40; 3.50)	1.00 to 18.00	4.00 to 18.00

Abbreviations: SAD, source‐to‐axis distance; WED, water equivalent depth.

^a^ About 20% of cases have tumor with widest dimensions up to 25 mm.

^b^ Distance tumor edge to optic disc <2 mm.

^c^ No strict indication.

**TABLE 3:** Dosimetrical characteristics of ocular PT centers included in this work.

	Distal dose fall‐off DDF 80% to 20% (g/cm^2^)		Lateral penumbra LP 80% to 20% (mm) Measured at the middle of the SOBP with a FS−25 mm^a^				Clinical protocol for SOBP normalization		
	Average over all combinations	Measurement tool	At entrance region	At middle SOBP	At distal region	Measurement tool	Protocol	Dose reference point for normalization	SOBP definition used for treatment
HZB‐Charite, Berlin	0.07	PTW Markus	1.40	1.60	1.80	IBA razor Diode	Modified IAEA TRS−398	Point at the middle of the SOBP	p90% to d90%
UFHPTI, Florida	0.32	IBA PPC05	1.40	1.30	1.70	Gafchromic film EBT3	IAEA TRS−398	Maximum value	NA
HollandPTC	0.20	PTW Advanced Markus	1.50	1.55	2.15	PTW microdiamond	ICRU Report No. 78	Average value within the treatment field	p90% to d90%
IFJ PAN, Krakow	0.12	PTW Markus	1.19	1.34	2.05	PTW Diode PR	Modified IAEA TRS−398	Point at the middle of the SOBP	p90% to d90%
CAL, Nice	0.10	Diode	0.60	0.90	1.30	Gafchromic film EBT3	Modified IAEA TRS−398	Point at the middle of the SOBP	p90% to d90%
CPT PSI, Villigen	0.11	NA	NA	NA	1.70–1.90	CCD camera	NA	NA	NA

Note

The results of the distal dose fall‐offs were averaged over all possible combinations between the range modulator wheels and the range shifter(s) values. For the lateral penumbra measurements, the SOBP width was chosen as close as technically possible to 3.00 g/cm^2^ in all the institutions.

Abbreviations: d, distal; DDF, distal dose fall‐off; FS, circular field size diameter; LP, lateral penumbra; p, proximal; SOBP, spread‐out Bragg peak.

^a^ A FS‐35 mm was considered at HollandPTC.

#### 2.3.3 Clinical simulation

Each of the six institutions used a different protocol for the SOBP normalization. A summary of the used protocols and their application is presented in the last three columns of Table 3. Three SOBPs were normalized according to all used protocol to demonstrate the differences among the protocols. A small SOBP (0.90 g/cm^2^), a medium SOBP (2.00 g/cm^2^), and a fully modulated SOBP (3.97 g/cm^2^) measured at HollandPTC were normalized (Table 4): (1) at their maximum values, (2) at the middle point of SOBPs, and (3) at the average value within the treatment field. Therefore, the choice of the protocol has an impact on the clinical definition of the SOBP region.

**TABLE 4:** Impact of the protocol on the SOBP width definition.

Protocol for SOBP normalization	Normalization	SOBP width (p90% to d90%)		
		Small SOBP width (0.90 g/cm^2^)	Medium SOBP width (2.00 g/cm^2^)	Full SOBP width (3.97 g/cm^2^)
IAEA TRS−398	Maximum value	0.873	1.985	3.939
Modified IAEA TRS−398	Point at the middle of the SOBP^a^	0.882	2.045	3.942
ICRU Report No. 78	Average value within the treatment field	0.893	2.004	3.944

Note

HollandPTC eyeline properties were considered.

Abbreviations: d, distal; p, proximal; SOBP, spread‐out Bragg peak.

^a^ Point defined as middle of p90% and d50%.

Additionally, depth dose distributions of seven different tumor scenarios were simulated to demonstrate the clinical impact of different eyeline characteristics. Three UM with different TH and LBD were defined as follows: Case 1, T1 UM: TH and LBD = 4 mm; Case 2, T2 UM: TH and LBD = 9 mm; and Case 3, T4 UM: TH = 15 mm and LBD = 22 mm. TH and LBD are surrogates for tumor volume definition. Estimated tumor staging reflected classification from the AJCC, updated Eighth edition.^17^ Commonly used clinical margins of 2.50 mm were applied.^3^, ^24^, ^44^, ^45^ The UM in Cases 1 and 2 was located anteriorly, at the equator, or posteriorly within the eye and in Case 3, only at the equator. For each clinical scenario, an isocentric treatment with a straight gazing angle was simulated. The straight gazing angle was chosen to highlight the differences from clinical configurations. The individual pristine Bragg peak of each center was combined with the HollandPTC library of Varian RMWs to create case‐specific SOBPs. With this approach, the impact of the different beam characteristics will be evaluated without creating a bias from different designs of the RMWs and normalizations.

## 3 RESULTS

### 3.1 Depth dose characterization

#### 3.1.1 Pristine Bragg peaks and energy spreads

**FIGURE 2:**
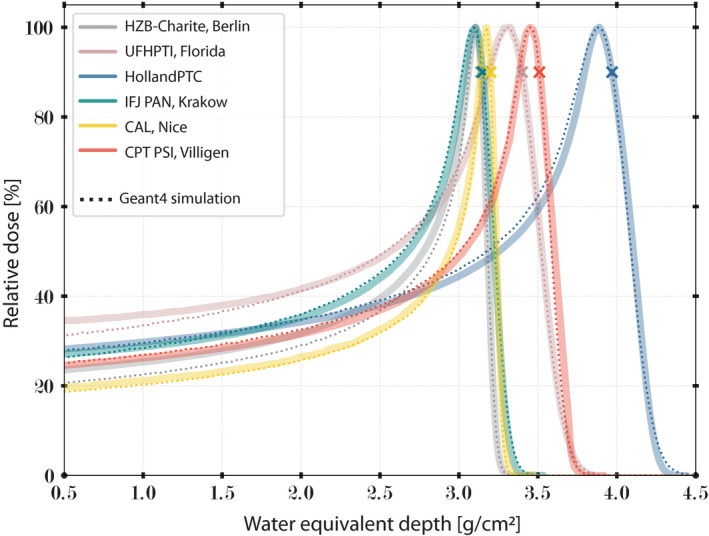
Geant4‐simulated energy spread determination. Interinstitutional comparison of the measured vs Geant4‐simulated pristine Bragg peaks with the maximal beam energy and normalized at their maxima. Commissioned range R_90%_ of every institution is marked by a cross. [Color figure can be viewed at wileyonlinelibrary.com]

The measured central‐axis pristine Bragg peaks were free of any beam modifying element in the beam path except the ionization chambers. Measured and Geant4‐simulated pristine Bragg peaks are shown in Figure 2. All Bragg peaks were normalized to the maximum dose values, and the curves were matched to each other at their maximum range in water R_90%_. The *x* axis defines the virtual WED.

At HollandPTC, the most distal pristine Bragg peak corresponded to a maximum WED range of 4.23 g/cm^2^ and a distal fall‐off of 0.18 g/cm^2^. The maximum WED range was commissioned with the unique scattering foil positioned into the beam path, equals 3.97 g/cm^2^. The accuracy of residual ranges at R_90%_ was within ±0.05 g/cm^2^. The average DDF was 0.18 g/cm^2^ (SD = 0.004 g/cm^2^). The range shifter thickness did not affect the DDF of the pristine beams. In comparison, DDF across centers were (g/cm^2^) (1) CAL, Nice: 0.057; (2) HZB‐Charite, Berlin: 0.064; (3) CPT PSI, Villigen: 0.087; (4) IFJ PAN Krakow: 0.107; and (5) UFHPTI, Florida: 0.186.

Based on Geant4 simulations, differences in energy spread values Δ*E* ^40^ linked to the individual pristine Bragg peaks were found to be (MeV) (1) CAL, Nice: 0.10; (2) HZB‐Charite, Berlin: 0.20; (3–5) CPT PSI, Villigen, IFJ PAN Krakow, UFHPTI, Florida: 0.70; and (6) HollandPTC: 1.10 MeV.

#### 3.1.2 Modulated ocular beams

**FIGURE 3:**
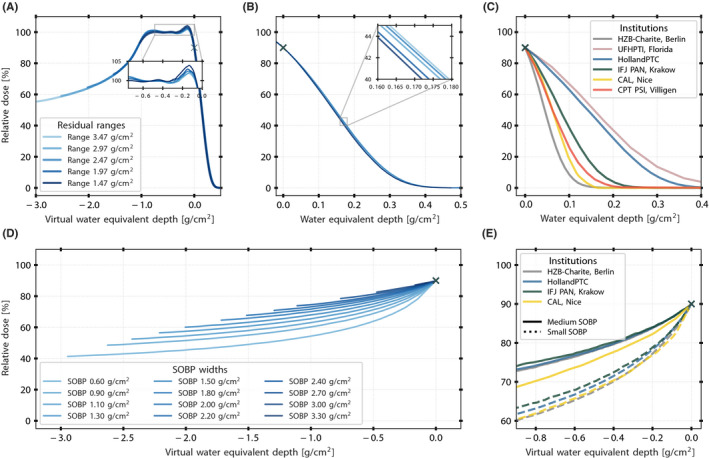
Distal and proximal characteristics of measured modulated and collimated beams. (A) Clinical spread‐out Bragg peaks (SOBPs) of 1.10 g/cm^2^ measured at HollandPTC with a FS‐35 mm for five residual ranges. (B) Zoomed‐in distal fall‐off for the same configurations. (C) Interinstitutional comparison of the distal fall‐off of fully modulated beams measured with a FS‐25 mm (a FS‐35 mm was considered at HollandPTC) and along the central axis of the proton beam. (D) Proximal dose distributions as a function of SOBP width measured at HollandPTC with a FS‐35 mm. (E) Interinstitutional comparison of the proximal region of small and medium modulation widths: small SOBP <1.00 g/cm^2^ in solid line and medium SOBP </> 1.00–2.00 g/cm^2^ in dashed line. Data were not available at UFHPTI, Florida, and CPT PSI, Villigen. [Color figure can be viewed at wileyonlinelibrary.com]

RMWs are required to create uniform depth dose distributions (clinical SOBPs) by spreading the pristine Bragg peak out in the longitudinal direction. The design of each wheel was defined by Monte Carlo simulations prior to the installation. The wheels are designed in a way to reach an optimal compromise between dose homogeneity in the treatment field and the sharpness of the distal fall‐off. A “hot spot shoulder” at the distal end of the SOBP improves the distal fall‐off but compromises the homogeneity. In total, 16 RMWs were commissioned to cover all possible clinical scenarios. Each variable range shifter value can be used only within a defined range. For example, a thicker range shifter is required for a given SOBP width and a shallow‐seated UM than for a posterior tumor. The impact of RMW on DDF in water varied from 0.206 (residual range of 3.67 g/cm^2^) to 0.197 g/cm^2^ (residual range of 1.17 g/cm^2^). Figure 3A shows an example of a modulation width of 1.10 g/cm^2^ as a function of residual range, and a zoomed‐in distal region is displayed in Figure 3B. The impact of the Varian RMWs on the surface doses is displayed in Figure 3D.

The DDFs of modulated beams across institutions including HollandPTC are shown in Figure 3C, and the values are presented in Table 3. As the DDF is not affected by the SOBP width, the DDFs were calculated from the SOBPs with full modulation. The curves were normalized according to the ICRU Report No. 78. The average DDF of 0.20 g/cm^2^ of the HollandPTC eyeline is right between the average DDF of 0.32 g/cm^2^ of UFHPTI, Florida,^42^ and the four other institutions. The DDF at CAL, Nice; HZB‐Charite, Berlin; IFJ PAN, Krakow; and CPT PSI, Villigen, was on average 50% sharper than at HollandPTC (Table 3). To illustrate the proximal behavior, a set of two clinical SOBPs with range shifter values equal to zero (small SOBP < 1.00 g/cm^2^ in dashed line, medium SOBP <> 1.00–2.00 g/cm^2^ in solid line) were used (Figure 3E). The differences in angular widths of the RMWs, energy spread, and low‐energy proton contamination resulted in a difference in the proximal dose.

### 3.2 Lateral dose characterization

**FIGURE 4:**
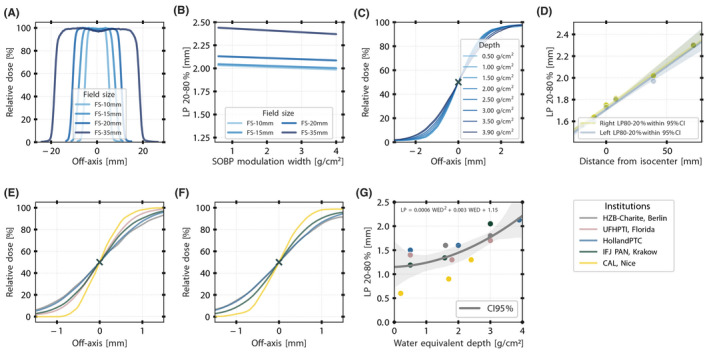
Lateral dose profiles of measured modulated and collimated beams. (A) Lateral profiles of the HollandPTC nozzle as a function of field size. All profiles were measured at the isocenter and middle of the fully modulated SOPB. (B) Dependency of the lateral penumbra on spread‐out Bragg peak (SOBP) modulation width of the HollandPTC nozzle. All profiles were measured at the isocenter and middle of each SOPB. (C) Dependency of the lateral profile of the HollandPTC nozzle on the point of measurement in water. All profiles were measured with a FS‐35 mm and the fully modulated SOBP at eight different depths. (D) Lateral penumbra dependency on air gap distance between the surface of the water phantom and the snout exit of the HollandPTC nozzle. All profiles were measured with a FS‐35 mm and the fully modulated SOBP. Zero corresponds to the isocenter. Confidence interval is given at 95%. (E) Interinstitutional comparison of the lateral profiles measured at the isocenter with a fully modulated SOBP. The SOBP width was chosen as close as technically possible to 3.00 g/cm^2^ in all the institutions. (F) Lateral profiles of a FS‐10 mm and of a FS‐20 mm (a FS‐25 mm was considered at UFHPTI, Florida). (G) Lateral penumbra LP 20% to 80% values as a function of the water equivalent depth and measured with a FS‐25 mm (a FS‐35 mm was considered at HollandPTC). Confidence interval is given at 95%. [Color figure can be viewed at wileyonlinelibrary.com]

Lateral dose profiles measured at HollandPTC are shown in the upper panel of Figure 4. The smaller the FS, the sharper the lateral fall‐off. For example, for a given SOBP modulation width of 1.10 g/cm^2^, lateral 80% to 20% penumbra is lowered by 0.40 mm when reducing a circular FS‐35 mm to a FS‐10 mm. Smaller SOBP modulation widths also correspond to larger lateral fall‐off values (Figure 4A and B). The lateral profile dependence on depth in water of the HollandPTC eyeline is shown in Figure 4C. In accordance with other published results,^42^, ^46^ the distance between the exit of the nozzle and the isocenter is typically 70 mm. This prevents dose distribution horns from edge scattering in the lateral profiles at closer distances and minimizes unwanted increase of the LP at larger distances. At HollandPTC, the lateral fall‐off at 50‐mm distance from the snout exit is reduced to 1.56 mm (Figure 4D), compared with 1.75 mm at the isocenter, with no horn profiles observed (Figure 4A).

The symmetry of the cross‐field profiles at the middle of the fully modulated SOBP was within 0.50% for circular field diameters from 10 up to 35 mm. Flatness increased as a function of FS, from 0.40% (FS‐10 mm) to 1.72% (FS‐35 mm).

LPs for circular FS‐10 mm and FS‐20 mm measured at the isocenter plane were compared across institutions (Figure). The dependence of the LP on depth in water within a 95% confidence interval is shown in Figure 4G. The LP for the same FS in water degrades slightly faster at HollandPTC compared with beamlines with longer virtual SAD (Table 2). This was explained by a larger beam divergence and a wider projected source size at the point of interest. However, the beam current may be comprised at the isocenter when the range shifter location is moved closer to the source to sharpen the lateral fall‐off. For example, at HZB‐Charite in Berlin, the LP is less steep than technically possible to achieve a robust and reproducible beam position (Figure 4E,F).

### 3.3 Absolute absorbed dose determination and neutron dose at HollandPTC

**FIGURE 5:**
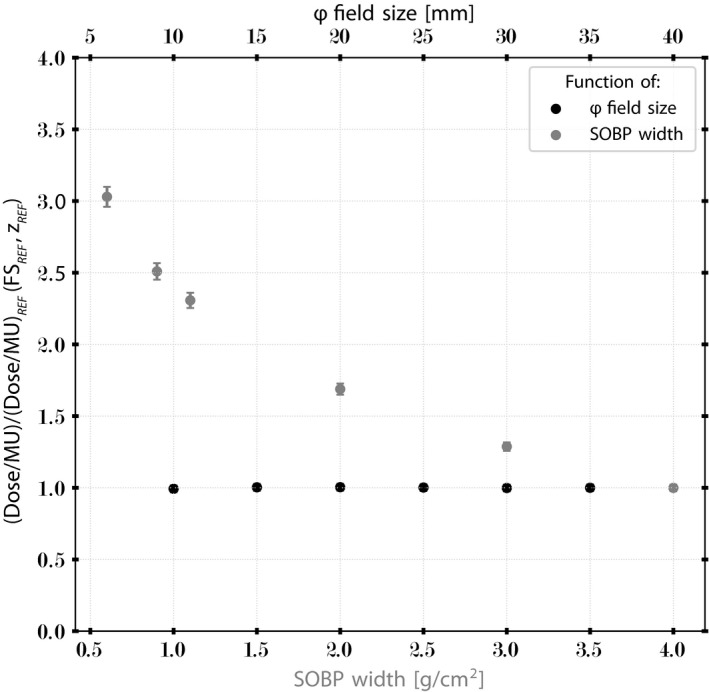
Dependency of output factor on field size and spread‐out Bragg peak modulation of the HollandPTC eye nozzle. Error bar function indicates 2.30% of measurement uncertainties. [Color figure can be viewed at wileyonlinelibrary.com]

The absorbed dose was determined under reference conditions (SOBP_REF_ = full modulation, FS_REF_ = 35 mm, z_REF_ = 1.50 g/cm^2^) as a function of FS and in‐depth modulation width. Figure 5 plots the ratio (Dose/MU)/(Dose/MU)_REF_(FS_REF_, z_REF_) for various FS diameters (10, 15, 20, 25, 30, and 35 mm) and SOBP modulation widths (ranging from 0.60 to 3.97 g/cm^2^). The Dose/MU quantity is a key parameter for clinical routine. No significant change in Dose/MU among different FS diameters was observed within 0.50%. A Dose/MU ratio of 3:1 was noted between the smallest clinical SOBP width of 0.60 g/cm^2^ and the full modulation of 3.97 g/cm^2^.

For neutron dose measurements, 500 MU under reference conditions were delivered. It corresponded to a proton dose of 0.97 Gy. The neutron doses were integrated over 3 s of measurement, and the results were 35.00, 9.20, and 7.80 µSv/Gy at the locations of the contralateral half brain, chest region, and abdominal region, respectively. For a prescribed treatment dose of 60 Gy(RBE), equivalent neutron doses to the three body locations corresponded to 2.16 ± 0.43, 0.55 ± 0.11, and 0.47 ± 0.09 mSv, respectively.

### 3.4 Translation to clinics

The differences in SOBP width definition when using different protocols are summarized in Table 4. The differences can be as large as 0.06 g/cm^2^ between IAEA TRS‐398 and modified IAEA TRS‐398 for a defined wheel. This difference is larger than the clinically accepted uncertainty of 0.05 g/cm^2^ defined at HollandPTC. The largest differences occurred for the medium SOBP width of a theoretical value of 2.00 g/cm^2^, ranging from 1.985 (IAEA TRS‐398) to 2.045 g/cm^2^ (modified IAEA TRS‐398).

**FIGURE 6:**
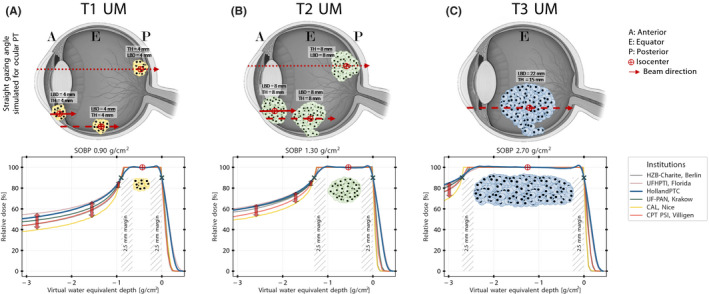
From technical differences to clinical discrepancies in terms of depth dose distributions: clinical simulations for three scenarios (AJCC staging^17^): (A) T1 UM, (B) T2 UM, and (C) T3 UM. Tumors were located anteriorly (ciliary body tumor), at the equator, or posteriorly. Tumor height (TH) and largest basal diameter (LBD) in straight gazing angle direction were used for spread‐out Bragg peak (SOBP) modulation. The dose was prescribed at the isocenter. Simulations were performed with the individual pristine Bragg peak of every institution and the HollandPTC library of range modulator wheels. SOBP region was defined between 90% distal to 90% proximal doses; 2.50 mm distal and proximal margins were used. [Color figure can be viewed at wileyonlinelibrary.com]

Figure 6 presents the results of the SOBP calculation for seven simulated clinical scenarios. The most considerable variations were in the proximal dose for a posterior pole located UM. For instance, the difference in proximal relative depth proton dose for an SOBP of 0.90 and 1.30 g/cm^2^ between CAL, Nice, and UFHPTI, Florida, reached up to 20% within the first 1.50 and 1.00 g/cm^2^ along the proton beam path, respectively.

## 4 DISCUSSION

In this study, the physical and dosimetrical properties of the first eyeline designed by Varian for ocular PT are reported and compared against several different ocular PT designs implemented worldwide. The differences in beam parameters are translated into differences in the depth dose distributions within the eye, demonstrating how different technical parameters can impact the plan quality. Albeit intercomparison studies among several PT centers have been published,^47^ centers that were built over the last decade are missing from this work of particular relevance. Moreover, no international guideline for ocular PT exists, and therefore, new centers are highly dependent on close collaboration with existing institutions and publications of the beam characteristics. The incidence of UM patients is estimated between one and nine cases per million population per year.^1^ The corresponding clinical demand for treating such ocular lesions by PT is currently covered by 20 institutions worldwide.^47^

The essential characteristics of ocular proton beams are LP and distal fall‐off, as well as a high‐dose rate. The LP is usually clinically more critical than the distal fall‐off as the tumor is often directly abutting ocular structures. However, to minimize irradiation of surrounding tissue, both lateral and distal penumbrae have to be as steep as possible. In order to mitigate various uncertainties of proton beam dose delivery in ocular patients, clinical margins are applied. Ocular PT has been remarkably successful with using a common 2.50‐mm margin, built upon a long‐standing international consensus in ocular PT.^3^, ^4^, ^5^, ^20^, ^22^, ^24^, ^44^ It appears robust against involuntary motion and setup uncertainties in all scenarios (gazing angles). Although technical differences leading to clinical discrepancies cannot be ignored as shown in this study, a high local tumor control of up to 95% is reported by all the institutions.^2^, ^3^, ^4^, ^5^, ^6^, ^7^, ^8^, ^9^, ^10^

The distal fall‐off is determined by the beam design properties and the initial energy leaving from the accelerator. The ideal scenario would be using a low‐energy cyclotron dedicated to the treatment of ocular lesions resulting in the sharpest distal penumbra for all possible combinations of the RMWs and the range shifter values like in HZB‐Charite, Berlin, and CAL, Nice. However, new centers are usually part of a multiroom PT institution with several gantries, and therefore, the initial extracted energy leaving the cyclotron is high (up to 250 MeV). In such cases, as the beam needs to be degraded down to 70–75 MeV before entering the ocular nozzle, the energy spread is much higher due to range straggling and limited transmission efficiency of the degrader.^48^ The use of beryllium for the degrader at low energies improves the proton transmission up to 28% by lowering the scattering effect compared with widely used carbon.^49^ The intrinsic nature of the degrader group and its compounds and the setting of the energy slit (energy width) explain the wider energy spread of 1.10 MeV occurring at the HollandPTC eye nozzle. In fact, both gantries, the research room, and the eye treatment room use the same energy selection system, and the slit settings affect the beam current passing through it.^48^ The resulting energy dispersion is an important factor determining the shapes of the pristine Bragg peaks, as shown in this study. The variation in the shape of the Bragg peak consequently leads to the differences in the distal fall‐off of clinical SOBPs. At HollandPTC, the average distal fall‐off of 0.20 g/cm^2^ for all clinical ocular situations is less steep than in other institutions, expect for the IBA eye nozzle at UFHPTI, Florida.

There is a way though to improve the distal fall‐off by adjusting the design of the RMWs. By optimizing the weighting of the last RMW steps, the distal fall‐off can be improved at the price of creating a dose “hot spot shoulder” at the end of the SOBP. The challenge is to find the right balance between a distal fall‐off steepness and a homogeneous dose across the SOBP or the treatment field. Increasing the former entails degrading the latter.

Related to this topic is the fact that there is no common consensus on how to normalize the clinical SOBP. Using either the IAEA TRS‐398 protocol *stricto sensu*, its modified version, or the ICRU Report, may result in different SOBP widths and treatment FSs. In this study, we showed that the chosen protocol had impacted the SOBP widths. The difference for HollandPTC example was up to 0.06 g/cm^2^. The increment between two consecutive SOBP widths is 0.30 g/cm^2^ or less (SOBP resolution); therefore, depending on the choice of the protocol, different RMWs could be selected for the treatment. This demonstrates the necessity of having a unified protocol.

The trade‐off between distal fall‐off and treatment irradiation time, which depends on the dose rate, is another challenging aspect that needs to be addressed in ocular PT. While commercial solutions, as the one from Varian presented in this study, provide one trade‐off over all combinations of RMWs with the range shifter values, some institutions have put substantial efforts towards in‐house developments to adjust this for individual treatment or groups of treatment. For instance, with OPTIS 2 at the Paul Scherrer Institute, *adaptive beam tuning* depending on the tumor case clustering^50^ has been proposed. One can argue that posterior‐seated tumors abutting the optic disc and/or macular area will suffer the most from a larger distal fall‐off than ciliary body or iris tumors where the proton dose drops inside the vitreous body. The near‐maximum dose (D_2%_) for the optic disc and macula will deteriorate the vision acuity irreversibly.^51^ Consequently, time matters the most for anterior tumors. The shorter irradiation times lead to fewer gazing motion uncertainties during a treatment fraction. For example, the energy slit settings at the Paul Scherrer Institute are adjusted to four opening scenarios from 12 to 20 mm, as commonly done with scanning systems.^40^ The CAL, Nice, center was initially designed for neutron treatments, and therefore, it is the only institute that can continuously modulate the beam current to maintain a treatment fraction within 10 s. The dose rate may go up to 100 Gy/min, which almost corresponds to flash treatment modalities.

The differences among institutions in dose in the proximal region are demonstrated in this study. In particular, the most considerable difference was identified in the dose distribution to T1 and T2 deep‐seated UMs. In a recent paper, Espensen et al.^51^ assessed the late radiation‐induced complications in a large cohort of choroidal tumor patients after ocular PT based on the dose delivered to healthy structures. Remarkably, proton dose differences of up to 20% in the proximal region among institutions, as shown in this study, may result in an increased risk of ocular hypertension and neovascular glaucoma, especially for T1 and T2 posterior‐seated choroidal tumors. For a prescribed RBE equivalent dose of 60 Gy, the RBE equivalent dose of 12 Gy received by the anterior segment will most likely increase ocular toxicities.^52^, ^53^, ^54^, ^55^ In line with Espensen et al., the dosimetrical properties of a new eyeline could be included in *a priori* toxicity prediction models.

To investigate the parameters influencing the LP, all institutions provided the measurements at the middle of the maximum SOBP. However, it was technically not possible to reach the same SOBP width due to the differences in the design of the RMWs among the institutions. The maximum difference in the fully modulated SOBP was 0.82 g/cm^2^ leading to an error on the LP of 0.02 mm estimated from the HollandPTC data. In general, the value of 2 mm for the LP is considered to be optimal. However, it is difficult to achieve such a sharp penumbra in ocular PT. The behavior of the lateral spread suffers considerably from changes in the geometry of the beam scattering system. Usually, a single scattering system results in the smallest penumbra. Additionally, preabsorbing materials as range shifters delivering the shallowest Bragg peaks (anterior tumors) scatter the beam more. If the air gap distance is not minimized to reduce scattering in air, it can lead to beam broadening. Reducing the energy directly from the high‐energy cyclotron itself could lower the LP, but it is not possible with multiroom setups. For horizontal dedicated ocular beamlines, it is more promising to optimize the distance. Geometry matters the most. The sharpness of the LP can be improved by an infinite source‐to‐axis distance (leading to a highly focused beam), a short distance between the range shifter and the patient, a reasonable length of the eye nozzle avoiding any additional proton deflection, and a small air gap between the patient‐specific aperture and the patient. The standard distance between nozzle exit and isocenter is 70 mm, which results from a compromise between the sharpness of the LP, room layout, and allowing the patient to stare at the light. This distance could eventually be minimized for a clipless workflow,^56^ as the space for the blinking light support would not be needed. Reducing the air gap by 20 mm leads to a gain of 0.20 mm in the LP, as shown in this study. Even a small gain has a clinical impact, as the LP is still the primary parameter to spare organs‐at‐risk.^57^ Another alternative could be the use of synchrotron‐based facilities.^15^, ^58^, ^59^, ^60^ Ciocca et al.^15^ reported that a constant pulsed proton beam with energy set to around 70 MeV without degrading the beam has led to LPs between 1.40 and 1.70 mm for various geometry‐shaped FSs between 6 and 25 mm. However, the reported treatment time was up to 3 min. Moreover, commercial solutions are rare.

In general, the passive scattering is limited by the fact that the systems by themselves do not provide perfect degraders (with no scattering) or perfect scatterers (with no energy loss). The simple scattering foil designed by Varian and clinically used at HollandPTC scatters the protons less efficiently than in the other institutions. The clinical advantage consists in the irradiation time that can be maintained close to or below 60 s for all the treatments, even for large FSs. However, the physics price to pay for using one unique scattering foil in clinics is the increased scattering due to its thickness. On the other hand, such a design is easy to use, less prone to clinical errors, less cumbersome to perform patient quality assurance, and overall, less costly. The Paul Scherrer Institute has an in‐house double scattering design for improved efficiency and less energy loss and spread. The first scatterer consists of the range shifter. The second scatterer is a so‐called multiple ring scattering system made out of three concentric rings; each ring is made of a combination of tantalum and plexiglass. By combining low‐Z and high‐Z materials, one can control both the multiple scattering and the energy loss over a finite range. Eventually, the lateral profile at the isocenter plane becomes homogenous within 2.50%. At IFJ PAN in Krakow, two sandwiched thin foils are used.^61^ The closer the scattering foil to the beam source, the sharper the lateral fall‐off for high‐energy cyclotron‐based systems. The average effective source distance measured at HollandPTC is 120 cm and leads to an effective source size of 4.40 mm.

Even though the detector used in this study is intended for radioprotection dosimetry, the estimation of the low‐energy secondary neutron doses absorbed by the contralateral half brain, chest, and abdominal regions lie within the order of magnitude of previously published data.^15^, ^42^ Neutron doses are noticeably lower at the chest and abdominal regions than at the contralateral half brain. It is important to note that the biological damage to the particular healthy surrounding tissues caused by fast neutron contamination remains unclear.

A limitation of the dosimetrical comparison study among institutions is that each institution used its own measurement devices and setups. The uncollimated pristine Bragg peak measurement may be noticeably affected by the detector used to measure the depth dose curves along the central axis, that is, diode versus Advanced Markus. A pristine Bragg peak measured with a diode accentuates the energy spread (wider) than the one measured with the Advanced Markus. Laterally, using Gafchromic films^62^ or diamond detectors also affects the results. Importantly, as the method for field‐specific and patient‐specific calibration measurements had not been standardized among institutions for this study, interinstitutional comparison of output factors with dedicated passive scattering eyelines is not reported here. Additionally, all the measurements were performed for FSs larger than 10‐mm diameters as usually only treatment diameters larger than 10 mm are irradiated. With the isotropic lateral margin of 2.50 mm, this consideration is sufficient for UMs presenting basal diameters larger than 5 mm. For smaller UMs, proper considerations regarding small‐field dosimetry should be taken into account.^63^

## 5 CONCLUSION

Based on this study, we can conclude that the dosimetrical properties of the ocular PT system from Varian fall within the range of the currently existing centers, and therefore, the same clinical concepts can be applied. This is an important finding as the excellent clinical outcome of ocular PT is highly dependent on the beam properties of the system. Furthermore, the interinstitutional comparison of this study could be used to define guidelines or recommendations that would help new centers to design and tune their ocular PT system.

## CONFLICT OF INTEREST

This research was cofunded by the research program PROTONS4Vision (Grant NWO 14654), which is financed by the Netherlands Organization for Scientific Research (NWO), Technology Foundation STW, the Top consortium for Knowledge & Innovation (TKI‐HTSM), and Varian Medical Systems Particle Therapy GmbH & Co. KG, Troisdorf, Germany.
